# Dynamic analysis of mother–preschooler interactions: Understanding its role in the associations between maternal emotion dysregulation and child psychopathology

**DOI:** 10.1017/S0954579426101886

**Published:** 2026-09-23

**Authors:** Salome Vanwoerden, Lauren M. Laifer, Emily Russell, Olivia A. Frigoletto, Amy L. Byrd, Maureen Zalewski, Stephanie D. Stepp

**Affiliations:** 1 Psychiatry, University of Pittsburgh, USA; 2 Psychology, University of Oregon, USA

**Keywords:** child psychopathology, dyadic dynamics, emotion dysregulation, emotion socialization, mother–child interactions

## Abstract

Theory and research highlight the role of maternal emotion socialization in the development of emotion dysregulation and associated psychopathology in offspring. This study described and quantified emotion socialization dynamics (i.e., mutual negativity and positivity) between mothers and their preschool-aged children, testing associations between dyadic dynamics and maternal emotion dysregulation and examining the predictive power of dyadic dynamics for symptoms of child psychopathology. Participants were 76 mother–child dyads (mothers: 22–46 years old, *M* = 33.17 (4.83); children 36–47 months old, *M* = 42.40 (3.78), 55.6% female, 47% racial/ethnic minoritized status) who completed frustration-eliciting and naturalistic play tasks. Child emotions and mothers’ emotion socialization behaviors were coded. Mutual negativity during play was positively associated with mother-rated child aggression (*β* = 0.25, *p* = .017), whereas mutual positivity during play was negatively associated with teacher-rated attention (*β* = 0.35, *p* = .001) and internalizing problems (*β* = −0.25, *p* = .011). Findings suggest that mother–child dynamics during play may have unique associations with child mental health symptoms and highlight the utility of examining emotion socialization as a dyadic process.

Emotion dysregulation is a transdiagnostic factor accounting for psychopathology risk across the lifespan (Beauchaine & Cicchetti, 2019). Research demonstrates positive associations between emotion dysregulation in parents and their offspring (Bridgett et al., 2015), suggesting that the intergenerational transmission of emotion dysregulation may contribute to the development of psychopathology among offspring (Ip et al., 2021). Links between parents’, and especially mothers’, emotion dysregulation and child psychopathology have been demonstrated for internalizing problems (Crespo et al., 2017) and a range of externalizing problems (Crespo et al., 2017), including aggression (Lau & Williams, 2022), and suicide (Doba et al., 2025). Given the robust link between maternal emotion dysregulation and child psychopathology, it is necessary to understand factors that may account for this association.

Emotion socialization (i.e., the process through which parents teach their children to identify, express, and regulate their emotions; Eisenberg et al., 1998) has garnered attention as a key mechanism influencing child emotion regulation and related socioemotional outcomes. In their foundational model, Eisenberg et al. (1998) propose that parents contribute to child emotion regulation through specific emotion-related socialization behaviors, including parental reactions to children’s emotions. Parents’ responses to children’s emotions represent salient opportunities for emotion socialization, with supportive responses (e.g., accepting, validating, comforting) predicting greater child emotion regulation and nonsupportive responses (e.g., punishing or dismissing) predicting more emotion *dy*sregulation (Perry et al., 2020) and symptoms of psychopathology (Frigoletto et al., 2024). Effective parental emotion regulation plays a crucial role in promoting adaptive parenting behaviors, such as supportive responses, and reducing nonsupportive responses (Edler & Valentino, 2024a). Accordingly, parents’ responses to children’s emotions may be a central process through which parents’ emotion dysregulation predicts offspring psychopathology characterized by emotion dysregulation.

The preschool stage (i.e., 3–4 years of age) is a period of rapid neurobiological and socioemotional development, when foundational self-regulatory capacities emerge (Carlson & Wang, 2007) yet children remain highly reliant on caregivers for external support and co-regulation (Calkins & Hill, 2007). As such, parental responses to child emotion may be particularly important during this period when regulatory skills are highly malleable (Bridgett et al., 2015; Morris et al., 2017). Notably, emotional and behavioral patterns adopted during the preschool stage often become characteristic (Cole et al., 1994), predicting psychopathology and poorer psychosocial functioning through adulthood (Arslan et al., 2021; Korhonen et al., 2018). Thus, research examining parental responses to children’s emotions as a malleable process, specifically during the preschool period, has the potential to inform early prevention and intervention efforts.

While most prior work on emotion socialization has focused primarily on parents’ behaviors, the parent–child relationship is an inherently dyadic process and should be studied as such (Moore et al., 2013; West et al., 2020). The dynamic systems perspective emphasizes dyads as complex systems, with characteristics distinguishable from the individual members of the dyad (Ram et al., 2014). Over repeated interactions, system behavior becomes more predictable, with frequently occurring patterns stabilizing (Lewis & Granic, 2000). Recurrent dyadic patterns become attractor states, a term reflecting states that dyads find themselves pulled into or “attracted to.” Attractor states can be understood and quantified using state space grids (SSGs) (Hollenstein, 2013), which plot the behavior of each member of a dyad onto a two-dimensional grid in temporal order, creating a visual map of the interaction trajectory (see Figure 1). Next, measures of attractor states can be calculated, including how long a dyad spends in an attractor state and how quickly the dyad enters this space (and, conversely, when they last leave the space). Importantly, when directly comparing predictive value of dynamic indicators of mother–child interactions to more static, or global, assessments, Coburn et al. (2015) found that prenatal maternal distress was uniquely associated with dynamic indicators of mother–infant interactions at 12-week postpartum. When accounting for the shared variance between dynamic and global indicators of the same interactions, global indicators were no longer associated with maternal distress, supporting the utility of more dynamic methods.

Two attractor states that have been found to be relevant in the context of emotion socialization are mutual negativity and mutual positivity, which represent joining in same-valence behaviors (Coburn et al., 2015). More specifically, capturing parent behavioral responses in the context of child emotion expression, rather than in isolation, is the essence of emotion socialization in real time. Mutual negativity, operationalized as states during which children’s negative emotion expressions co-occur with parents’ nonsupportive responses, has been associated with maternal depression (Coburn et al., 2015; Roben et al., 2015), albeit not consistently (Connell et al., 2011), and may predict increased internalizing symptoms in preschool-aged children (Guo et al., 2021).

While studied less frequently, accumulating research points to the importance of the socialization of positive emotions (Breaux et al., 2022), operationalized with the attractor state of mutual positivity (i.e., child positive affect co-occurring with supportive parental responses). For example, studies using similar dynamic operationalizations suggest that positive emotion socialization may be impaired by maternal depression (Coburn et al., 2015; Hummel et al., 2016), although more research is needed on how positive emotion socialization is associated with offspring outcomes. Given that the bulk of previous research has focused on maternal depression, additional research moving beyond single categorical disorders is needed. Focusing on maternal emotion dysregulation as a transdiagnostic indicator can provide broader implications for understanding the relation between positive and negative emotion socialization and symptoms of child psychopathology (Zitzmann et al., 2024).

Importantly, it is possible that the effects of these two attractor states may have differential associations when observed across contexts (e.g., positive, playful contexts versus more challenging contexts that elicit frustration for the dyad). Different experimental tasks elicit various forms of mother–child behavior (Caron et al., 2006; Donenberg & Weisz, 1997) and maternal behavior across contexts may have different origins and functions (Bozicevic et al., 2025; Leerkes et al., 2012). Some research on other parenting constructs suggests that parents’ responses in distressing contexts may be more predictive of children’s emotion regulation and wellbeing than those in nondistressing contexts (Bozicevic et al., 2025; Leerkes et al., 2012); however, other theories suggest that parent behavior in positive contexts like play facilitates socialization processes (Grusec & Davidov, 2010). However, it remains to be determined whether dynamic interaction patterns observed in challenging or distressing versus nondistressing or play contexts demonstrate different associations with maternal emotion dysregulation and child outcomes.

Our first aim was to describe and quantify the dynamics of emotion socialization (i.e., mutual negativity and positivity) between mothers and their preschool-aged children and to examine their associations with maternal emotion dysregulation. We examined mother–child interactions across two salient contexts of the preschool period: naturalistic play and a frustration-inducing task. Second, we evaluated the predictive power of these dynamics for symptoms of internalizing problems, aggression, and attention problems, after accounting for maternal emotion dysregulation. Given past research linking maternal emotion dysregulation with both maternal socialization behaviors and child psychopathology, controlling for maternal emotion dysregulation was important to understand whether any associations observed were merely an artifact of broader maternal emotion dysregulation. Furthermore, while our data were cross-sectional, limiting our ability to test any mechanistic associations between these variables, this statistical design would fit theoretical models of the role of emotion socialization in how maternal emotion dysregulation impacts child psychopathology. We included both mother and teacher reports of child psychopathology symptoms given robust evidence that while these reports are not always aligned (Martel et al., 2017), they present complementary sources of information regarding child functioning (Hoffmann et al., 2023). While aggression and attention problems are sometimes evaluated as existing in the same spectrum of externalizing problems, previous research has identified differential predictors for these types of externalizing psychopathology (Frigoletto et al., 2024). We hypothesized that for dyads in which mothers report higher emotion dysregulation, interactions across all contexts would be characterized by stronger attractor states of mutual negativity and weaker mutual positivity attractors. Consistent with previous research (e.g., Bozicevic et al., 2025), we expected that mutual negativity during the frustration task would uniquely predict all forms of child psychopathology. Given the relative lack of research on positive emotion socialization, we had no directional hypotheses about associations between mutual positivity and symptoms of child psychopathology.

## Methods

### Participants and procedures

The current sample was drawn from one site of a multi-site randomized controlled trial of Dialectical Behavior Therapy (DBT) for mothers of preschool-age (36–47 months old) children. Participants included *N* = 90 mother–child dyads recruited using a university-based research recruitment program and by contacting psychiatric treatment clinics. To achieve greater variability in maternal emotion dysregulation, mother–child dyads were oversampled for maternal symptoms of borderline personality disorder. Specifically, to be included in the sample, mothers had to exhibit at least 3 symptoms of borderline personality disorder, which had to include either the affective instability or anger criteria and the interpersonal dysregulation criterion. Given the broader goals of the study, this ensured that the sample had adequate representation of the features that DBT is designed to treat. *n* = 14 dyads were not included in the current analysis due to not having observational coding data available.^1^.

Therefore, the total sample of mother–child dyads included in the current analysis was N = 76. Mothers ranged in age from 22.24 to 45.61 years (*M*_age_ = 33.17, *SD* = 4.83; 35% racial/ethnic minoritized status) and offspring ranged in age from 36.10 to 47.77 months (*M*_age_ = 42.4, *SD* = 3.78; 55.6% female; 47% racial/ethnic minoritized status). Dyads were included if mothers were biological parents with at least 50% physical custody of the child from birth and were excluded if children had a developmental delay. Both mothers and children demonstrated standardized scores of at least 70 on measures of verbal IQ Peabody Picture Vocabulary Test Fourth Edition (Dunn & Dunn, 2007) and the Expressive Vocabulary Test Second Edition (Williams, 2012).

Participants completed a baseline laboratory visit prior to being randomized to treatment. All data used in the current analysis were collected during this baseline visit; thus, treatment was not considered as a variable in this manuscript. Mothers provided contact information for their child’s preschool teacher or daycare provider. Providers were contacted and asked to complete an online questionnaire assessing broadband psychopathology as observed in the preschool/daycare setting. Mother–child dyads completed a series of semi-structured interactions during which they were audio and videotaped. Videos were watched and coded after the fact (see description below).

### Measures

#### Child psychopathology outcomes: Child Behavior Checklist (CBCL) and Teacher Report Form (TRF)

The CBCL/TRF is a broadband measure of parent- and teacher-reported psychopathology. All items are rated on a scale from 0 (*not at all true*) to 2 (*very true*). Item responses are summed for a total score with higher scores representing greater problems. This measure is widely used and has demonstrated adequate psychometric properties (Achenbach, 2018). For the current study, we used the scales for Internalizing Problems (parent report: *α* = 0.92, 36 items; teacher report: *α* = 0.84, 32 items), Aggression Problems (parent report: *α* = 0.94, 19 items; teacher report: *α* = 0.95, 25 items), and Attention Problems (parent report: *α* = 0.83, 5 items; teacher report: *α* = 0.88, 9 items).

#### Maternal emotion dysregulation: Difficulties in Emotion Regulation Scale (DERS)

The DERS is a 36-item self-report measure of perceived emotion regulation in which mothers rate how often each item applies to them on a 5-point Likert scale ranging from 1 (*almost never*) to 5 (*almost always*). The DERS captures six subscales of emotion dysregulation: lack of emotional awareness, lack of emotional clarity, impulse-control difficulties, difficulties engaging in goal-directed behaviors, nonacceptance of emotional responses, and limited access to emotion regulation strategies. Items were summed to create a total score, with higher scores indicating greater levels of emotion dysregulation. Cronbach’s alpha for this scale was 0.97 in the current sample.

#### Dynamic indicators of mutual negativity and mutual positivity

##### Mother–child interaction tasks.

Mothers and children were videotaped during two consecutive interaction tasks, each of which lasted 5 minutes. First, dyads completed a Lego Figure Task, intended to elicit frustration. Dyads were shown a photograph of a difficult Lego sculpture that the child was instructed to build. A key Lego piece for the sculpture was missing, and mothers were told they could verbally help the child but not touch the Legos. Next, dyads engaged in Free Play, intended to approximate naturalistic play. Interactions were videotaped, and maternal behaviors and child emotion were coded after the fact.

***Observational coding*** of videotaped interactions used a coding manual developed for the current study, which is included in the Online Supplement. A team of undergraduates and staff was trained and supervised by the first author and met on a weekly basis to minimize coder drift. All coding was completed using Datavyu 1.3.8 (Datavyu Team, 2014), an open-source academic software that allows users to view multiple data streams while simultaneously making timestamped annotations, and automates data extraction and analysis. For all behaviors, onset and offset times were recorded, creating a continuous coding system. Interrater reliability was evaluated by having two coders independently score *n* = 35 cases (46% of the sample). Matching codes were identified if the onset or offset times were within a 1,000 ms window of each other, and based on Cohen’s Kappa, interrater reliability was excellent (Maternal Responses = 0.93, Child Emotion = 0.90) as calculated in EasyDIAg software (Easy Determination of Interrater Agreement; Holle & Rein, 2015). EasyDIAg is another open-source toolbox developed to assist researchers in calculating interrater reliability for timed-event sequential data by using an algorithm to match ratings of the same event or unit. Prior to analysis with SSGs (see below), the time series of codes were broken into 2-s intervals for analysis.

***Maternal nonsupportive responses*** included hostility (expressions of irritation or frustration with the child that are critical or rejecting) and dismissal (extent to which mother seems uninvolved with or unresponsive toward the child, ignoring or not fully acknowledging child’s emotional experience/minimizing).

***Maternal supportive responses*** included positive responses (responding to the child in a positive, affectionate, or reinforcing way), validation (communicating that child’s emotional experience is appropriate, acceptable, or understandable), and comforting. Neutral states were coded in the absence of any indicators of nonsupport or support.

***Child emotional expressions*** included **positive** and **negative** emotions as indicated by posture, facial expressions, vocal tone, and content of speech. Neutral states were coded if there were no clear emotion indicators.

##### Mutual negativity and mutual positivity.

We operationalized two synchronous behavior-emotion states using multiple indicators that were calculated using SSGs in Gridware 1.15a (Lamey et al., 2004). SSGs were created separately for each task by mapping maternal behaviors and child emotion expressions onto a 3 × 3 grid with the *X*-axis representing maternal emotion socialization behaviors (supportive, neutral, nonsupportive) and the *Y*-axis representing child emotion (positive, neutral, negative). For each 2-s interval that behaviors and affect were coded, a node was plotted on the grid, and consecutive nodes were connected to create a dyadic trajectory plot (see Figure 1).

We calculated several indicators: (a) **total duration**: the total time a dyad spent in the synchronous behavior-affect space; (b) **number of visits** to the synchronous behavior-affect space, with the number of visits operationalized as any time that the dyad entered into this space from any other region on the SSG; (c) **first entry**: the amount of time from the start of the interaction until the first time the dyad entered into the synchronous behavior-affect space; and (d) **last exit**: latency of time until the dyad exited each synchronous behavior-affect space for the last time before the end of the interaction. Stronger attractor states were indicated by longer total durations, greater number of visits, shorter amount of time before the first entry into the space, and longer time until the dyad’s last exit out of the space.

Because each of these indicators provide similar, but non-overlapping information about attractor state strength and due to the overall small sample size, we conducted a dimension reduction technique, or principal component analysis (PCA), to extract a single indicator of attractor strength. Thus preliminary analyses using these four variables in each of the four sets of attractor states (mutual negativity and positivity) and contexts (play and frustration) were conducted and are described in detail below.

#### Potential covariates

Variables related to maternal emotion dysregulation, mother–child interaction dynamics, and child psychopathology were evaluated for potential inclusion to models. Child age (in months) was included in models based on research showing that maternal sensitivity increases as children age (Perea-Velasco et al., 2023) and that similarly, there are increases in psychopathology onset with age (Roberts et al., 1998). Child biological sex has been associated with maternal emotion socialization behaviors (Klimes-Dougan et al., 2007) as well as rates and presentation of different forms of psychopathology (Hartung & Lefler, 2019). Whether child identified with a minoritized ethnic or racial group was also included, with previous research demonstrating differences in maternal socialization behaviors and psychopathology presentation based on ethnoracial identity (Morelen & Thomassin, 2013; Winstead & Sanchez-Hucles, 2019). Lastly, maternal age (in years) was included due to implications for family dynamics with increasing maternal age (Ahmad et al., 2024). Lastly, we included a variable indicating whether the family received public assistance as a proxy for socioeconomic status (SES) given previous research suggesting higher SES associated with more supportive emotion socialization behaviors (Shaffer & Egeland, 2011) and lower psychopathology risk (Winstead & Sanchez-Hucles, 2019).

### Data analytic strategy

Prior to running analyses, the four indicators for each of the two attractor states of mutual positivity and negativity were examined within and across tasks using descriptive statistics, bivariate correlations, and t-tests (four separate analyses for: mutual positivity during play, mutual negativity during play, mutual positivity during frustration, mutual negativity during frustration). PCAs with varimax rotation were run to reduce the number of variables in primary analyses given the small sample size as indicators were expected to be strongly correlated while also providing unique information about respective attractor states. Extraction based on eigenvalues >1 was conducted with resulting scores used in primary analysis. Tables S3–S10 and Figures S1–S4 in the online supplement list eigenvalues, factor loadings, and scree plots for each of these four analyses. Results from all four PCAs revealed a single component for each analysis explained between 76.52% (mutual positivity during play) to 86.69% (mutual negativity during frustration task) of variance in the four respective indicators with high factor loadings ≥ |0.78|. These four components were named as either mutual positivity or negativity for their respective tasks. Component scores were computed using the regression method, yielding weighted linear composites of the standardized indicators; resulting scores were centered at zero, with variances equal to the corresponding component eigenvalues.

Next, descriptive statistics were calculated to examine the distributions of and bivariate correlations between main study variables. During this step we also evaluated the associations between potential covariates and primary variables. Only covariates that were significantly correlated with both outcome and independent variables were included in subsequent analyses. For the first aim of evaluating associations between maternal emotion dysregulation and mutual negativity and positivity (across contexts), we applied Bonferroni corrections for multiple tests (*p* = .05/4 correlations = 0.0125) to evaluate statistical significance.

Lastly, we conducted a series of four separate regression analyses predicting child psychopathology as reported on by parents and teachers (separately) across frustrating and play contexts. Multiple correlated dependent variables (internalizing, attention, and aggression problems) were included in each regression to account for the correlated nature of psychopathology. As independent variables, maternal emotion dysregulation and both mutual negativity and positivity of each context were included (see Figure 2). Regressions were analyzed using Mplus 8.10 (Muthén & Muthén, 1998). 95% confidence intervals (CIs) were calculated to evaluate reliability of effects.

All data and analysis code for this project are available on OSF (https://osf.io/umyv5/overview).

## Results

Prior to running primary analyses, we evaluated the 16 individual attractor state indicators. Table S1 lists statistics for the frequency distributions of and bivariate correlations between each of these indicators. We conducted paired samples *t*-tests to evaluate differences between indicators of different attractor states within the same task as well as between the same indicators across different tasks. Mutual positivity was a stronger attractor state in both the frustration and play tasks, with differences medium in strength (absolute value of Cohen’s *d* = 0.01–0.72; Table S2) suggesting that, overall, our sample was more likely to be pulled into mutual positivity, regardless of the context. No differences between indicators of attractor state strength across tasks were statistically significant; however, attractor strength of mutual negativity was slightly higher during frustration vs. play tasks (absolute value of Cohen’s *d* = 0.17–0.22).

Individual indicators of each attractor state within the two tasks were highly correlated (within-state and task correlations = −0.65–0.98; Table S1), with a single component for each set of indicators accounting for a large amount of the variance (see results above). Thus, including resulting PCA components as independent variables in primary analyses allowed us to maximize information while minimizing the overall number of variables tested in our small dataset. The resulting variables (4 total) represented strength of each attractor state (mutual positivity and negativity) during each context (play and frustration) that were used in subsequent analyses.

Descriptive statistics for all main study variables are listed in Table 1 alongside bivariate correlations. Receipt of public assistance was positively related to most reports of child psychopathology and maternal emotion dysregulation; thus, it was included in subsequent analyses as a covariate. Bivariate correlations between maternal emotion dysregulation and child psychopathology revealed consistent, moderately sized correlations with all mother-rated forms of psychopathology, but only with teacher-rated Internalizing Problems. Maternal emotion dysregulation was also associated with stronger mutual negativity during the play task. However, it was not associated with any other attractor states, and once we applied Bonferroni correction for multiple comparisons, the association was no longer statistically significant.

Mutual negativity during the frustration task was associated with greater mother-rated Internalizing Problems, whereas mutual negativity during play was associated with both greater mother-rated Attention and Aggression Problems. While mutual positivity during the frustrating task was not associated with mother-rated child psychopathology, mutual positivity during play was negatively associated with fewer teacher-rated Attention and Internalizing Problems. All these correlations were small to medium in size (Cohen, 1988).

Next, we conducted multiple regressions to identify unique associations between attractor states and child psychopathology, while accounting for levels of maternal emotion dysregulation and receipt of public assistance. First, we examined the context of play (Table 2). In the regression model predicting mother-rated psychopathology, mutual negativity during play had a unique positive association with Aggression Problems. In contrast, in the model predicting teacher-rated psychopathology, mutual positivity predicted fewer Attention and Internalizing Problems. Mirroring bivariate results, maternal emotion dysregulation positively predicted all forms of mother-rated psychopathology.

In the set of regressions predicting child psychopathology from behavior during the frustrating task (Table 3), dyadic behavior did not uniquely contribute to the prediction of mother-rated or teacher-rated child psychopathology outcomes. Again, maternal emotion dysregulation was associated with mother-rated psychopathology as well as teacher-rated Internalizing Problems.

In contrast to bivariate correlations revealing an association between maternal emotion dysregulation and mutual negativity during play, there were no significant correlations between maternal emotion dysregulation and dyadic behavior in the regression models. Furthermore, attractor states were not correlated.

## Conclusions

This study evaluated associations between maternal emotion dysregulation and mother–child emotion socialization dynamics across contexts, and how they were associated with child psychopathology. Maternal emotion dysregulation was largely not associated with dyadic behavior, suggesting mutual positivity and negativity were truly dyadic in nature. While maternal emotion dysregulation was robustly associated with mother-rated psychopathology symptoms in preschoolers, it only predicted teacher-rated internalizing problems. On the other hand, dyadic behavior during play had consistent associations with both mother- and teacher-rated internalizing and externalizing problems, whereas dyadic behavior during the frustrating task was not associated with these same child outcomes. Specifically, after accounting for severity of maternal emotion dysregulation, mutual negativity was associated with greater mother-rated aggressive behavior, whereas mutual positivity was associated with fewer teacher-rated attention and internalizing problems. Results point to the importance of understanding context-specific, dynamic dyadic patterns of interaction to understand early presentations of child psychopathology.

Analyses revealed that dyadic attractor states were uniquely correlated with child psychopathology when observed during play but not in a more frustrating context. Sample-level descriptives suggested that the frustration task may have elicited slightly more negative dyadic dynamics relative to the play task, though this was not statistically significant. Thus, expressions of mutual negativity may have been more atypical during the play task, and stronger attractor states of mutual negativity in a context not designed to elicit negative emotions (i.e., play) may be uniquely associated with more adverse outcomes. Indeed, we found that stronger attractor states of mutual negativity during play were associated with higher levels of mother-rated aggressive behavior. The role of mutually coercive cycles for the development of aggression is well established (Granic & Patterson, 2006), and our previous work has demonstrated that child physiological reactivity during parent–child conflict predicts aggression in daily life (Byrd et al., 2022, 2025). The current study extends this work by suggesting that dyadic dynamics between mother and child – not merely either partner’s individual contribution – may play an important role in the development of aggression.

Because mutual negativity in this study captured the behavior of both dyad members, we also considered whether it could be attributable to individual differences in mothers’ own emotion dysregulation: although maternal emotion dysregulation and mutual negativity were associated at the bivariate level, this was likely explained by their shared associations with child aggression, suggesting that mutual negativity is not simply a proxy for maternal dysregulation. At the same time, given the cross-sectional nature of our design, it is possible that mutual negativity during play was driven more by child characteristics than mothers’ (e.g., Taraban & Shaw, 2018). Together, these findings underscore the need to evaluate emotion socialization as a dyadic, dynamic process, rather than the product of either partner alone.

Stronger mutually positive attractor states during naturalistic play were related to lower levels of attention and internalizing problems as observed by teachers, aligning with previous research using similar methodology (Guo et al., 2021). This finding builds on the small but growing literature regarding the importance of enhancing positive emotion socialization to improve child outcomes (Breaux et al., 2022) and theory suggesting that parental behavior in non-distressing contexts may foster reciprocity and learning in offspring (Grusec & Davidov, 2010). Additionally, unstructured play contexts may provide greater opportunities for parent–child dyads to foster and build on these positive emotion dynamics, which can enhance offspring positive affect (Hill et al., 2023) and serve as a model for positive reciprocity that youth may apply in novel social contexts (Grusec & Davidov, 2010). For example, a behavioral parent training intervention that teaches parents to coach children during friendship interactions and encourages parent behaviors that foster a positive emotional climate improved internalizing problems among youth with attention-deficit/hyperactivity disorder (Smit et al., 2022).

A large body of research has identified maternal emotion dysregulation as a risk factor for offspring psychopathology across the internalizing-externalizing spectrum (Frigoletto et al., 2024; Lau & Williams, 2022; Price & Kiel, 2022), with preliminary evidence pointing to causal effects of maternal emotion dysregulation on externalizing problems in offspring (Byrd et al., 2021). Thus, it was not surprising that maternal reported emotion dysregulation was associated with all forms of psychopathology. However, this finding was not robust across raters, with non-significant associations between maternal emotion dysregulation and teacher-rated externalizing problems (both attention and aggression problems).

Rater discrepancy of child psychopathology is a known phenomenon, partly due to different expressions of behavior across settings and different perceptions of the threshold for problem behavior (Rescorla et al., 2014). For example, in our sample, mother-rated attention problems were significantly lower than teacher-rated attention problems, suggesting greater salience of these issues in the school context. In contrast, mothers rated child aggression and internalizing problems higher than teachers did. Previous findings demonstrate that parental psychopathology may predict parents’ perspectives of youth psychopathology (Clark et al., 2017; Vanwoerden et al., 2023); thus, in the present study, maternal emotion dysregulation may contribute to elevated maternal ratings of child aggression and internalizing problems in addition to the effect of shared method bias.

Surprisingly, while findings in the current sample (Byrd et al., 2023; Frigoletto et al., 2024) and extant research (Edler & Valentino, 2024a) have identified associations between maternal emotion dysregulation and maternal responses to children’s emotion, we found minimal associations between maternal emotion dysregulation and the dyadic attractor states investigated. Notably, although maternal emotion dysregulation was associated with more mutual negativity during play at the bivariate level, these did not survive corrections for multiple comparisons or reach statistical significance in the regression models, suggesting that associations between emotion dysregulation and dyadic attractor states may be explained by their shared association with child psychopathology. Alternatively, our sample size was small, which could contribute to statistically insignificant findings, especially given that dyadic dynamics were operationalized using a relatively novel, but understudied method (observational coding and SSGs). Previous research examining associations between maternal emotion dysregulation and emotion socialization behaviors (particularly mothers’ response to child emotions) have relied on self-reports of these behaviors (Edler & Valentino, 2024b), and even when using observational coding, mothers’ behaviors are coded in isolation. While our findings highlight the importance of studying dynamics as a dyadic phenomenon rather than being driven by characteristics of any single partner, future research should evaluate how dyadic attractor state strength is associated with previously used operationalizations of emotion socialization.

Future research should additionally evaluate how the combination of mother and child characteristics predicts different dyadic dynamics to understand how trait-level factors contribute to momentary dyadic dynamics (Hajal & Paley, 2020). Additionally, it is possible that attractor states not explored in this study are more relevant to emotion dysregulation. The focus on this study was on attractor states representing a joining of behaviors and emotions of similar valence, which could function to amplify individual behaviors. However, unexplored attractor states, for example, negative emotion-related socialization behaviors responses and child positive emotions (e.g., Song et al., 2019) may also be related to maternal emotion dysregulation. Additional work should investigate additional attractor states that represent misalignment between mother and child.

The current findings should be interpreted with consideration to their limitations. First, this study was interrupted by the COVID pandemic; thus, the full sample size was not achieved, which reduced statistical power and precluded our ability to run more complex analyses. To address this limitation, we used dimension reduction techniques to derive single estimates of attractor state strength to use in analyses, and we calculated 95% CI intervals to evaluate reliability of effects. Given that CIs for statistically insignificant effects were quite wide, we cannot rule out the possibility of associations between maternal emotion dysregulation, dyadic dynamics, and symptoms of child psychopathology. However, this study provides preliminary evidence of associations that should be followed up in future studies. Second, the cross-sectional and correlational nature of our design limited our ability to evaluate temporal effects of these constructs. Given the bidirectional nature of dyadic dynamics, it is highly likely that aspects of child psychopathology contribute to transactions between mother and child, as well as maternal emotion dysregulation. Lastly, our sample included only biological mothers despite the important role of non-birthing parents and caregivers in family dynamics and the development of psychopathology in children.

Despite these limitations, the current study provides the first evidence of differential associations of dyadic attractor states across contexts with child psychopathology. Findings highlight the importance of studying mother–child interactions as dynamic dyadic states as well as considering implications of the context in which these are displayed. Furthermore, findings suggest that maternal emotion dysregulation is not unilaterally associated with certain forms of dyadic dynamics, and additional research is needed to understand how mother–child interactions unfold and explore possible protective factors to support prevention efforts in at-risk dyads.

## Supplementary Material

Online Supplement

The supplementary material for this article can be found at https://doi.org/10.1017/S0954579426101886.

## Figures and Tables

**Figure 1.**
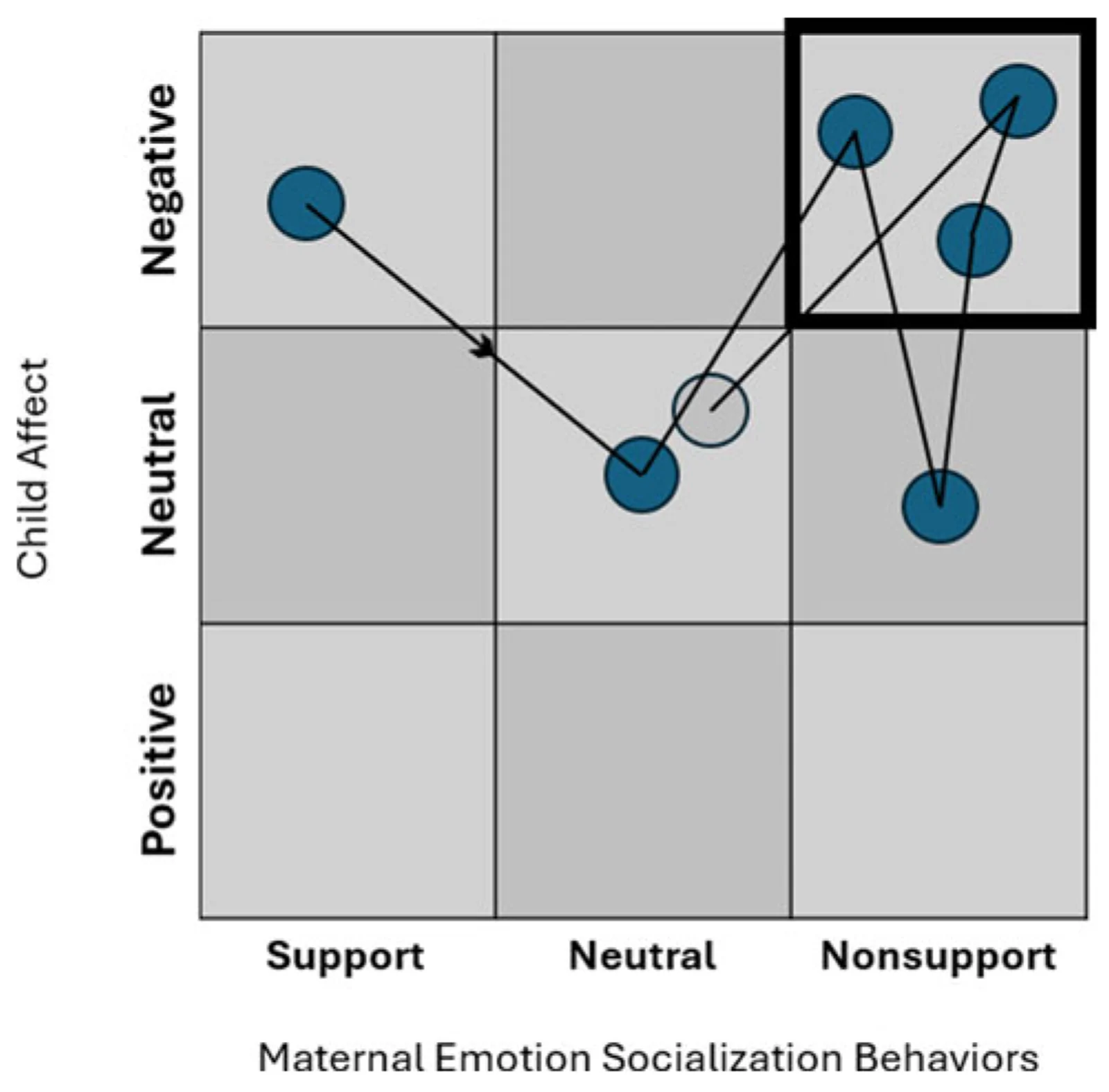
State space grid representing hypothetical mother–child interaction.

**Figure 2.**
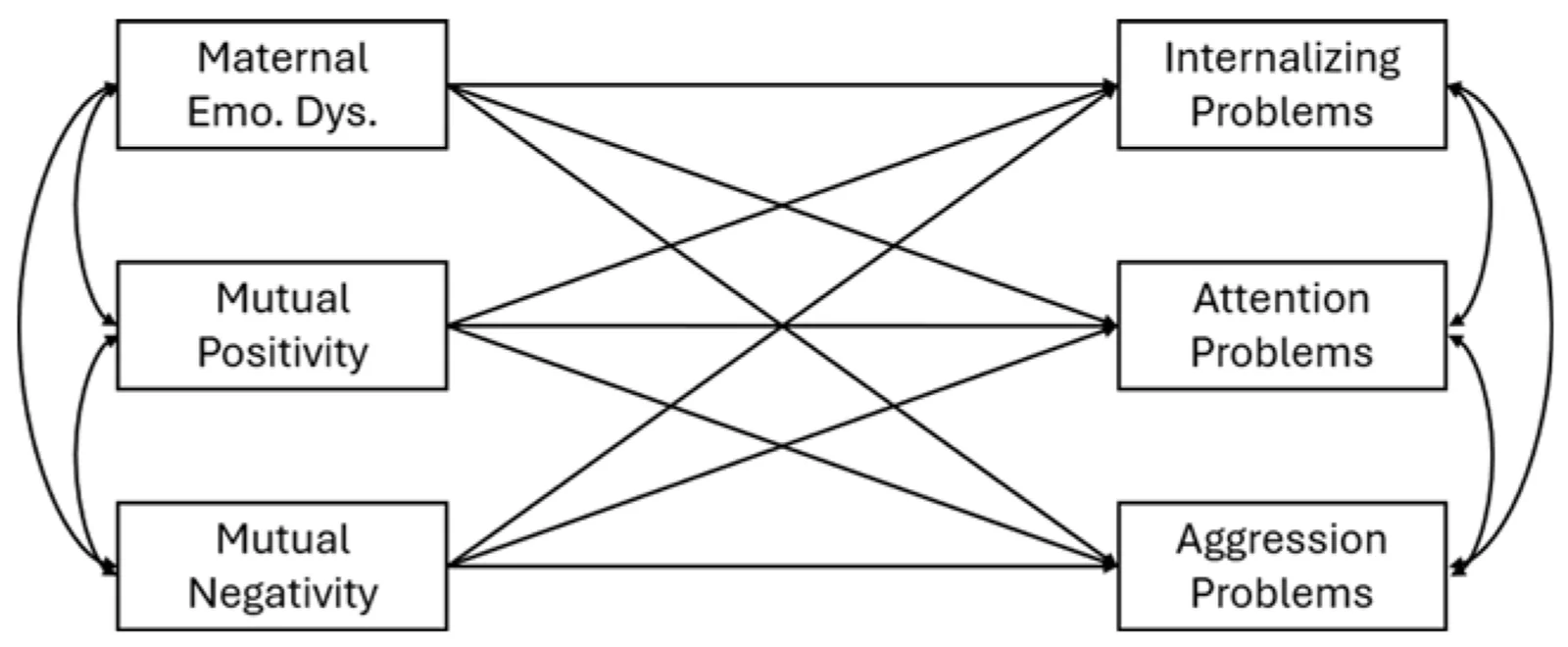
Regression models predicting child psychopathology from maternal emotion dysregulation and dyadic attractor states.

**Table 1.** Descriptive statistics and bivariate correlations between study variables

	1	2	3	4	5	6	7	8	9	10	11	12	13	14	15
1. CBCL attention															
2. CBCL aggressive	0.776**														
3. CBCL internalizing	0.658**	0.723**													
4. TRF attention	0.290*	0.292*	0.333**												
5. TRF aggressive	0.234*	0.383**	0.285*	0.719**											
6. TRF internalizing	0.378**	0.502**	0.414**	0.667**	0.737**										
7. DERS	0.421**	0.453**	0.434**	0.113	0.232	0.329**									
8. Lego: MN	0.114	0.083	0.270*	0.117	0.092	−0.022	0.138								
9. Lego: MP	−0.133	−0.123	−0.12	−0.000	0.162	0.054	−0.091	−0.012							
10. Play: MN	0.309**	0.321**	0.165	0.067	0.103	0.141	0.253*	−0.090	−0.120						
11. Play: MP	−0.133	−0.187	−0.135	−0.365**	−0.200	−0.283*	−0.074	0.100	0.201	0.006					
12. Child age	−0.043	−0.045	0.078	0.044	−0.105	0.058	−0.240*	0.073	0.070	−0.168	−0.145				
13. Child sex	−0.154	−0.084	−0.124	−0.115	−0.025	−0.096	−0.146	−0.062	0.056	−0.118	−0.041	0.069			
14. Child race	0.251*	0.234*	0.218*	0.182	0.153	0.083	0.125	0.164	0.048	0.014	−0.223	−0.055	0.209*		
15. Mom age	−0.200	−0.116	−0.278*	−0.081	−0.105	−0.018	−0.190	−0.072	0.140	−0.108	−0.152	0.054	0.218*	−0.099	
16. Pub. assist.	0.188	0.313**	0.226*	0.303*	0.296*	0.284*	0.257*	0.185	0.138	−0.020	−0.090	−0.054	−0.030	0.261*	0.111
Mean (SD)	2.45 (2.47)	11.24 (8.78)	8.84 (8.87)	4.17 (3.97)	6.89 (8.47)	6.45 (5.50)	85.81 (32.96)	0.00 (1.00)	0.00 (1.00)	0.00 (1.00)	0.00 (1.00)	41.53 (3.70)			33.13 (4.83)
Skew	1.22	1.03	2.38	0.62	1.63	1.04	0.38	3.35	1.10	4.11	0.79	0.12			0.06
Kurtosis	1.05	0.37	6.93	−0.65	2.48	0.47	−1.10	12.77	0.88	17.14	0.06	−1.37			−0.43
Range	0–10	0–34	0–48	0–13	0–36	0–22	36–158	−0.35–5.49	−0.82–3.35	−0.25–5.67	−0.93–2.75	36.10–47.77			22.24–45.61

Note.

* *p* < .05,

** *p* < .01.

CBCL = Child Behavior Checklist; TRF = Teacher Report Form; DERS = Difficulties in Emotion Dysregulation Scale; MN = mutual negativity; MP = mutual positivity. With Bonferroni-adjusted *p*-values (.0125) for correlations between DERS (maternal emotion dysregulation) and dyadic attractor states (Lego: MN, Lego: MP, Play: MN, Play: MP), none of these four correlation coefficients met threshold for statistical significance.

**Table 2.** Regression predicting child psychopathology by maternal emotion dysregulation and dyadic behavior during play task

Mother report (CBCL)												
	Attention				Aggression				Internalizing			
	Unsth. Est (*SE*)	95% CI	Stnd. Est.	*p*	Unsth. Est (*SE*)	95% CI	Stnd. Est.	*p*	Unsth. Est (*SE*)	95% CI	Stnd. Est.	*p*
Mutual negativity	0.55 (0.29)	[−0.02, 1.12]	0.22	.060	**2.18 (0.92)**	**[0.39, 3.98]**	**0.25**	**.017**	0.63 (1.22)	[−1.76, 3.02]	0.07	.606
Mutual positivity	−0.24 (0.25)	[−0.72, 0.24]	−0.10	.323	−1.27 (0.97)	[−3.17, 0.63]	−0.14	.190	−0.85 (0.65)	[−2.12, 0.42]	−0.10	.187
Emotion dysregulation	**0.03 (0.01)**	**[0.01, 0.04]**	**0.33**	**.003**	**0.09 (0.03)**	**[0.01, 0.04]**	**0.32**	**.006**	**0.10 (0.03)**	**[0.05, 0.16]**	**0.38**	**<.001**
Public assistance	0.53 (0.52)	[−0.48, 1.55]	0.11	.303	4.11 (1.89)	[−0.48, 1.55]	0.23	.030	2.24 (1.95)	[−1.59, 6.06]	0.13	.252
Teacher report (TRF)												
	Attention				Aggression				Internalizing			
	Unsth. Est (*SE*)	95% CI	Stnd. Est.	*p*	Unsth. Est (*SE*)	95% CI	Stnd. Est.	*p*	Unsth. Est (*SE*)	95% CI	Stnd. Est.	*p*
Mutual negativity	0.44 (0.34)	[−0.23, 1.11]	0.11	.196	0.88 (0.62)	[−0.34, 2.10]	0.10	.157	0.55 (0.56)	[−0.54, 1.64]	0.10	.325
Mutual positivity	**−1.38 (0.42)**	**[−2.21, −0.56]**	**−0.35**	**.001**	−1.46 (0.85)	[−3.11, 0.20]	−0.17	.085	**−1.38 (0.55)**	**[−2.45, −0.31]**	**−0.25**	**.011**
Emotion dysregulation	−0.01 (0.02)	[−0.04, 0.03]	−0.04	.733	0.03 (0.04)	[−0.05, 0.11]	0.25	.437	0.04 (0.02)	[−0.01, 0.08]	−0.20	.080
Public assistance	2.22 (0.93)	[0.40, 4.04]	0.28	.017	4.25 (2.04)	[0.25, 8.25]	0.12	.037	2.15 (1.32)	[−0.44, 4.74]	0.23	.103

*Note*. The bold values represent *p* < .05.

CBCL = Child Behavior Checklist; CI = confidence interval; SE = standard error; TRF = Teacher Report Form.

**Table 3.** Regression predicting child psychopathology by maternal emotion dysregulation and dyadic behavior during frustrating task

Mother report (CBCL)												
	Attention				Aggression				Internalizing			
	Unstd. Est (*SE*)	95% CI	Stnd. Est.	*p*	Unstd. Est (*SE*)	95% CI	Stnd. Est.	*p*	Unstd. Est (*SE*)	95% CI	Stnd. Est.	*p*
Mutual negativity	0.10 (0.21)	[−0.31, 0.51]	0.04	.638	−0.25 (0.76)	[−1.73, 1.24]	−0.03	.745	1.67 (2.22)	[−0.50, 3.84]	0.19	.132
Mutual positivity	−0.26 (0.25)	[−0.74, 0.23]	−0.10	.302	−1.11 (0.80)	[−2.66, 0.45]	−0.13	.164	−0.86 (0.80)	[−2.42, 0.70]	−0.10	.280
Emotion dysregulation	**0.03 (0.01)**	**[0.01, 0.04]**	**0.38**	**<.001**	**0.10 (0.03)**	**[0.04, 0.16]**	**0.38**	**.001**	**0.10 (0.03)**	**[0.05, 0.15]**	**0.37**	**<.001**
Public assistance	0.49 (0.56)	[−0.61, 1.59]	0.10	.384	4.23 (2.14)	[0.04, 8.43]	0.24	.048	1.95 (2.33)	[−2.63, 6.52]	0.11	.404
Teacher report (TRF)												
	Attention				Aggression				Internalizing			
	Unstd. Est (*SE*)	95% CI	Stnd. Est.	*p*	Unstd. Est (*SE*)	95% CI	Stnd. Est.	*p*	Unstd. Est (*SE*)	95% CI	Stnd. Est.	*p*
Mutual negativity	0.25 (0.38)	[−0.49, 1.00]	0.06	.504	0.34 (0.91)	[−1.44, 2.12]	0.04	.709	−0.42 (0.38)	[−1.17, 0.33]	−0.08	.273
Mutual positivity	−0.12 (0.58)	[−1.25, 1.01]	−0.03	.839	1.29 (1.11)	[−0.88, 3.46]	0.15	.243	0.42 (0.71)	[−0.98, 1.82]	0.08	.553
Emotion dysregulation	0.00 (.02)	[−0.03, 0.03]	0.01	.930	0.05 (0.04)	[−0.02, 0.12]	0.20	.167	**0.05 (0.02)**	**[0.01, 0.10]**	**0.31**	**.022**
Public assistance	2.37 (1.02)	[0.38, 4.36]	0.30	.020	3.56 (2.35)	[−1.05, 8.16]	0.21	.130	3.56 (2.35)	[−0.68, 4.95]	0.19	.130

*Note*. The bold values represent *p* < .05.

CBCL = Child Behavior Checklist; CI = confidence interval; SE = standard error; TRF = Teacher Report Form.

## Data Availability

All data and analytic code are available here: https://osf.io/umyv5/overview?view_only=7b0ad6380e1947729052c3acaa860bbc.

## Footnotes

1 *n* = 4 did not have videotapes of mother–child interactions, either due to a technical problem or because they withdrew consent to be videotaped, and *n* = 10 were recruited during a time when all in-person procedures were prohibited due to the COVID pandemic.
