# Supplementary material for: Dynamic analysis of mother–preschooler interactions: Understanding its role in the associations between maternal emotion dysregulation and child psychopathology
Source: Dev Psychopathol. Author manuscript; Available in PMC 2026 Nov 11. (PMC13753621; doi:10.1017/S0954579426101886)
Supplement: Supplementary file 1 [file NIHMS2221240-supplement-Online_Supplement.docx]

**Supplementary Materials**

Vanwoerden, S., Laifer, L., Russell, E., Frigoletto, O., Byrd, A. L., Zalewski, M., & Stepp, S. D. Dynamic analysis of mother-preschooler interactions: Associations between maternal emotion dysregulation and child psychopathology.

**Table of Contents**

a. **Table S1.** *Descriptive statistics and bivariate correlations between indicators of individual attractor states.*
b. **Table S2.** *Across task and state comparisons of the four indicators of attractor strength*
c. **Table S3**. *Eigenvalues and variance explained for principal component analysis on mutual negativity during the frustration task*
d. **Figure S1.** *Scree plot for principal component analysis on mutual negativity during the* frustration task
e. **Table S4.** *Component matrix for the single extracted component of mutual negativity during the frustration task*
f. **Table S5**. *Eigenvalues and variance explained for principal component analysis on mutual positivity during the frustration task*
g. **Figure S2.** *Scree plot for principal component analysis on mutual positivity during the frustration task*
h. **Table S6.** *Component matrix for the single extracted component of mutual positivity during the frustration task*
i. **Table S7**. *Eigenvalues and variance explained for principal component analysis on mutual negativity during the play task*
j. **Figure S3.** *Scree plot for principal component analysis on mutual negativity during the play task*
k. **Table S8.** *Component matrix for the single extracted component of mutual negativity during the play task*
l. **Table S9**. *Eigenvalues and variance explained for principal component analysis on mutual positivity during the play task*
m. **Figure S4.** *Scree plot for principal component analysis on mutual positivity during the play task*
n. **Table S10.** *Component matrix for the single extracted component of mutual positivity during the play task*
o. **Appendix**. *Description of observational codes*

**Table S1.** Descriptive statistics and bivariate correlations between indicators of individual attractor states.

| **Frustration:**  **Mutual Negativity** | Duration | # Visits | 1^st^ Entry | Last Exit |  | **Frustration:**  **Mutual Positivity** | Duration | # Visits | 1^st^ Entry | Last Exit |
| --- | --- | --- | --- | --- | --- | --- | --- | --- | --- | --- |
| # Visits | .97 |  |  |  |  | # Visits | .98 |  |  |  |
| 1st Entry | -.70 | -.81 |  |  |  | 1st Entry | -.71 | -.74 |  |  |
| Last Exit | .84 | .86 | -.76 |  |  | Last Exit | .76 | .75 | -.60 |  |
|  |  |  |  |  |  |  |  |  |  |  |
| Mean | 0.87 | 0.32 | 137.61 | 12.04 |  | Mean | 1.46 | 0.93 | 104.88 | 43.24 |
| Std. Dev. | 3.05 | 1.02 | 35.13 | 33.61 |  | Std. Dev. | 2.19 | 1.37 | 54.87 | 51.62 |
| Skew | 5.19 | 4.55 | -2.70 | 2.84 |  | Skew | 1.96 | 2.23 | -0.727 | 0.587 |
| Kurtosis | 31.93 | 25.06 | 5.87 | 7.26 |  | Kurtosis | 3.982 | 6.27 | -1.04 | -1.36 |
| Range | 0-22 | 0-7 | 1-150 | 0-146 |  | Range | 0-10 | 0-7 | 0-150 | 0-146 |
|  |  |  |  |  |  |  |  |  |  |  |
| Component Matrix | 0.94 | 0.98 | -0.87 | 0.93 |  | Component Matrix | 0.96 | 0.97 | -0.84 | 0.85 |
|  |  |  |  |  |  |  |  |  |  |  |
| **Play:**  **Mutual Negativity** | Duration | # Visits | 1^st^ Entry | Last Exit |  | **Play:**  **Mutual Positivity** | Duration | # Visits | 1^st^ Entry | Last Exit |
| # Visits | .96 |  |  |  |  | # Visits | .91 |  |  |  |
| 1st Entry | -.69 | -.83 |  |  |  | 1st Entry | -.65 | -.70 |  |  |
| Last Exit | .74 | .81 | -.48 |  |  | Last Exit | .70 | .73 | -.40 |  |
|  |  |  |  |  |  |  |  |  |  |  |
| Mean | 0.17 | 0.08 | 145.04 | 5.09 |  | Mean | 1.55 | 0.88 | 102.07 | 44.11 |
| Std. Dev. | 0.79 | 0.32 | 21.94 | 22.39 |  | Std. Dev. | 2.06 | 1.11 | 54.11 | 51.97 |
| Skew | 6.02 | 4.39 | -4.65 | 5.01 |  | Skew | 1.62 | 1.75 | -0.58 | 0.71 |
| Kurtosis | 41.01 | 20.54 | 21.54 | 26.55 |  | Kurtosis | 2.37 | 3.91 | -1.31 | -1.05 |
| Range | 0-6 | 0-2 | 18-150 | 0-148 |  | Range | 0-9 | 0-5 | 4-150 | 0-149 |
|  |  |  |  |  |  |  |  |  |  |  |
| Component Matrix | 0.94 | 1.00 | -0.83 | 0.84 |  | Component Matrix | 0.94 | 0.96 | -0.78 | 0.81 |

*Note.* Variance in all of the 4 indicators explained in by a single component: Frustration: Mutual Negativity = 86.70%; Frustration: Mutual Positivity = 81.98%; Play: Mutual Negativity = 81.75%; Play: Mutual Positivity = 76.52%.

**Table S2.** Across task and state comparisons of the four indicators of attractor strength

| **Mutual Negativity** | Frustration | Play | *t* (df=75) | *p* | Cohen's d |
| --- | --- | --- | --- | --- | --- |
| Total duration | 0.87 (3.05) | 0.17 (0.79) | 1.90 | .061 | 0.22 |
| Number of visits | 0.32 (1.02) | 0.08 (0.32) | 1.89 | .063 | 0.22 |
| First entry | 137.61 (35.13) | 145.04 (21.94) | -1.51 | .135 | -0.17 |
| Last exit | 12.04 (33.61) | 5.09 (22.39) | 1.45 | .152 | 0.17 |
|  |  |  |  |  |  |
| **Mutual Positivity** | Frustration | Play | *t* (df=75) | *p* | Cohen's d |
| Total duration | 1.46 (2.19) | 1.55 (2.06) | -0.30 | .766 | -0.03 |
| Number of visits | 0.93 (1.37) | 0.88 (1.11) | 0.29 | .770 | 0.03 |
| First entry | 104.88 (54.87) | 102.07 (54.11) | 0.36 | .721 | 0.04 |
| Last exit | 43.24 (51.62) | 44.11 (51.97) | -0.11 | .913 | -0.01 |
|  |  |  |  |  |  |
| **Frustration** | Mutual Negativity | Mutual Positivity | *t* (df=75) | *p* | Cohen's d |
| Total duration | 0.87 (3.05) | 1.46 (2.19) | -1.32 | .190 | -0.15 |
| Number of visits | 0.32 (1.02) | 0.93 (1.37) | -3.12 | .003 | -0.36 |
| First entry | 137.61 (35.13) | 104.88 (54.87) | 4.64 | <.001 | 0.53 |
| Last exit | 12.04 (33.61) | 43.24 (51.62) | -4.28 | <.001 | -0.49 |
|  |  |  |  |  |  |
| **Play** | Mutual Negativity | Mutual Positivity | *t* (df=75) | *p* | Cohen's d |
| Total duration | 0.17 (0.79) | 1.55 (2.06) | -5.42 | <.001 | -0.62 |
| Number of visits | 0.08 (0.32) | 0.88 (1.11) | -6.06 | <.001 | -0.70 |
| First entry | 145.04 (21.94) | 102.07 (54.11) | 6.31 | <.001 | 0.72 |
| Last exit | 5.09 (22.39) | 44.11 (51.97) | -5.94 | <.001 | -0.68 |

**Table S3**. Eigenvalues and variance explained for principal component analysis on mutual negativity during the frustration task

| Component | Total | % of Variance | Cumulative % |
| --- | --- | --- | --- |
| 1 | 3.47 | 86.69 | 86.69 |
| 2 | 0.33 | 8.19 | 94.88 |
| 3 | 0.19 | 4.68 | 99.56 |
| 4 | 0.02 | 0.44 | 100.00 |

**Figure S1.** *Scree plot for principal component analysis on mutual negativity during the frustration task*

**Table S4.** Component matrix for the single extracted component of mutual negativity during the frustration task

|  | Mutual Negativity |
| --- | --- |
| Total Duration | .944 |
| Number of Visits | .978 |
| First Entry | -.873 |
| Last Exit | .927 |

**Table S5**. Eigenvalues and variance explained for principal component analysis on mutual positivity during the frustration task

| Component | Total | % of Variance | Cumulative % |
| --- | --- | --- | --- |
| 1 | 3.28 | 81.98 | 81.98 |
| 2 | 0.41 | 10.21 | 92.19 |
| 3 | 0.30 | 7.37 | 99.55 |
| 4 | 0.02 | 0.45 | 100.00 |

**Figure S2.** *Scree plot for principal component analysis on mutual positivity during the frustration task*

**Table S6.** Component matrix for the single extracted component of mutual positivity during the frustration task

|  | Mutual Positivity |
| --- | --- |
| Total Duration | .959 |
| Number of Visits | .965 |
| First Entry | -.836 |
| Last Exit | .854 |

**Table S7**. Eigenvalues and variance explained for principal component analysis on mutual negativity during the play task

| Component | Total | % of Variance | Cumulative % |
| --- | --- | --- | --- |
| 1 | 3.27 | 81.75 | 81.75 |
| 2 | 0.52 | 13.07 | 94.82 |
| 3 | 0.21 | 5.14 | 99.96 |
| 4 | 0.00 | 0.05 | 100.00 |

**Figure S3.** *Scree plot for principal component analysis on mutual negativity during the play task*

**Table S8.** Component matrix for the single extracted component of mutual negativity during the play task

|  | Mutual Negativity |
| --- | --- |
| Total Duration | .944 |
| Number of Visits | .997 |
| First Entry | -.828 |
| Last Exit | .836 |

**Table S9**. Eigenvalues and variance explained for principal component analysis on mutual positivity during the play task

| Component | Total | % of Variance | Cumulative % |
| --- | --- | --- | --- |
| 1 | 3.06 | 76.52 | 76.52 |
| 2 | 0.60 | 15.00 | 91.52 |
| 3 | 0.25 | 6.28 | 97.80 |
| 4 | 0.09 | 2.20 | 100.00 |

**Figure S4.** *Scree plot for principal component analysis on mutual positivity during the play task*

**Table S10.** Component matrix for the single extracted component of mutual positivity during the play task

|  | Mutual Negativity |
| --- | --- |
| Total Duration | .939 |
| Number of Visits | .961 |
| First Entry | -.778 |
| Last Exit | .807 |

**Appendix**. *Description of observational codes*

The observational coding system was developed for the current sample. Below are direct excerpts from the coding manual. All behaviors were coded by marking the onset and offset that they were observed. Child emotional expressions were re-coded to either positive (happy) or negative (sad, mad, anxious). Maternal supportive (positive, validation) and nonsupportive responses (hostility, detachment) were combined for analysis.

**Child Emotional Expressions.**

Continuously coded (mutually exclusive) with neutral affect being the absence of any emotion code; if multiple emotions expressed, code the most intense/longest duration. allow 2 seconds to pass after overt affect expression before turning to neutral if nothing is overtly happening in the video. Can code child affect through points when mom is talking or researcher is talking. Note that there are points when a child may be experiencing an affect and something else happens (somebody enters the room or someone starts talking) that may ‘interrupt’ the child’s experience of emotion. This is an appropriate time to end an affect code if it is clear that the emotion has either switched or stopped.

- **(h) Happy**
  - Postural: Little tension in body, relaxed shoulders and chest, arms raised in excitement
  - Facial: Smiling, smooth forehead, upturned corners of mouth, crinkling in corners of eyes
  - Vocal: Light, lilting tone, higher and/or louder pitch, laughter, humming
  - Note: this code may be observed when children are playing with toys and are participating in ‘make believe’ (e.g., speaking in place of a toy animal). For this to be coded as positive affect, the child must be animated and engaged.
- **(s) Sad: glumness or tearfulness, disappointment**
  - Postural: Drooping head, slumped shoulders and/or body, eye rubbing to mask tears
  - Facial: Lip corners pulled down, quivering or loose/protruding bottom lip, drooping eyes, oblique-shaped brow (raised inner brows), tearfulness
  - Vocal: Lowered volume, whining vocalizations without protest
- **(m) Mad, angry: hostility, irritation, annoyance, frustration, or harshness**
  - Postural: Arms akimbo, finger wagging or jabbing
  - Facial: Furrowed brow, eyes narrowed, clenched or set jaw, pressed or tightened lips, tightened and narrow eyelids
  - Vocal: Harsh vocal tone, louder and/or deeper pitch, whining vocalizations with protest quality
  - Note: this code includes mild instances of frustration or annoyance
- **(a) Anxious & worry**
  - Postural: Jittery, repeated hand or foot movement, stiff upper body, raised shoulders
  - Facial: Furrowed brow, raised eyelids, lips retracted, lip-biting, darting glances
  - Vocal: Strained or stressed voice, shaky or tight tone
  - Note: this is a relatively uncommon code; it must be clear that the child is worrying or anxious about something. To distinguish this code from sadness, it can be helpful to consider whether the child is upset about something happening in the future (anxious) or in the past (sad); however, this distinction is not universally applicable but may be helpful in some instances when it is not clear whether sadness or anxiety is being displayed.

**Maternal Responses to Child Emotions**

Continuous coding, mutually-exclusive.

- **(p) Positive response:** responding to the child in a positive, affectionate, or reinforcing way: “Good job!” “Yes, you’re right.” “Wow!” “It’s ok.”; **accompanied by smiling, hugging or clear adding of warmth**. There must be a clear emotional component to the response, versus just saying “there we go” in a neutral tone (unless the praise is explicit such as “good job” or “good boy”). This code includes praising the child, responding in an emotionally warm way, or reinforcing the correctness of the child’s behavior (e.g., “good job!”). Positive responses are often accompanied by positive emotions or physical affection. Positive responses *are not* affirmative responses to the child (e.g., if the child asks if she is doing it right and mom says yes). Rather, positive responses are clear praises or reinforcements that are **unsolicited by the child**.
  - Note that this can be expressed non-verbally
- **(h) Hostility/Negativity:** verbal and non-verbal expressions of irritation or frustration with the child that are critical, or rejecting; includes teasing
  - if response is both hostile and dismissing, code hostility and not dismissing
  - tone is critical in determining hostility
- **(d) Detachment or Dismissing:** extent to which parent seems uninvolved with and unresponsive toward the child; ignoring child’s bids or not a full acknowledgement of child’s affective experience (minimizing). For example, if a child complains that the task is too hard, mom may say “it’s fine”
- **(v) Validation/Support:** any communication by mother that a child’s affective experience is appropriate, acceptable, or understandable.
  - Note that validation can be functional (mom helping fix the electrodes if child is complaining about them hurting) or verbal (saying ‘I’m sorry’ if child is expressing sadness)
  - While validation is typically coded after child expresses some affect, you can code ‘preemptive’ validation if there is a big event and mom responds quickly (before child can even react) to soothe—for example the child falls off chair or the Lego figure falls apart after lots of work.
